# High frequency and unique subtypes of meningioma in patients with *BAP1* tumor predisposition syndrome

**DOI:** 10.1007/s11060-026-05445-2

**Published:** 2026-02-11

**Authors:** Kaylee A. Ramsey, Lindsey Byrne, Olivia B. Taylor, Amr Soliman, Emma Schreiner, Isabella Gray, Alicia Latham, Rania Sheikh, Saman S. Ahmadian, Russell R. Lonser, Joshua D. Palmer, Maria I. Carlo, Colleen M. Cebulla, Mohamed H. Abdel-Rahman

**Affiliations:** 1 Havener Eye Institute, Department of Ophthalmology and Visual Sciences, The Ohio State University Wexner Medical Center, 400W 12th Ave, Room 225 Wiseman Hall, Columbus, OH 43210 USA; 2 Division of Human Genetics, Department of Internal Medicine, The Ohio State University Columbus, Columbus, OH USA; 3 Department of Medical Oncology, Memorial Sloan Kettering Cancer Center, New York, NY USA; 4 Department of Medicine, Memorial Sloan Kettering Cancer Center, New York, NY USA; 5 Department of Pathology, The Ohio State University Wexner Medical Center, Columbus, OH USA; 6 Department of Neurological Surgery, The Ohio State University Wexner Medical Center, Columbus, OH USA; 7 Department of Radiation Oncology, The Ohio State University Wexner Medical Center, Columbus, OH USA; 8 Present Address: Department of Pathology, The MetroHealth System, Cleveland, OH USA

**Keywords:** BRCA associated protein-1, BAP1, BAP1 tumor predisposition syndrome, BAP1-TPDS, Meningioma

## Abstract

**Purpose:**

*BAP1*-tumor predisposition syndrome (*BAP1*-TPDS) is associated with four main cancers: uveal melanoma, cutaneous melanoma, malignant mesothelioma, and renal cell carcinoma. However, additional cancers are found more rarely in *BAP1*-TPDS patients. The aim of this study was to investigate the association, clinical, and pathologic characteristics of meningioma in *BAP1*-TPDS.

**Methods:**

We conducted a retrospective chart review of meningiomas in two independent cohorts of patients with germline *BAP1* pathogenic or likely pathogenic (P/LP) variants at The Ohio State University Wexner Medical Center and at the Memorial Sloan Kettering Cancer Center from October 1st, 2010 date to April 21st, 2025. Additionally, we conducted a literature review of meningioma case studies for individuals with germline *BAP1* (P/LP) variants.

**Results:**

In a cohort of 237 subjects with *BAP1*-TPDS, we identified 6.8% (16/237) with a history of meningiomas. The average age of diagnosis was 44.5 years (17–71). For patients with available pathology, 61.5% (8/13) of the tumors were grade 2/3. Patients with available tumor tissue 83.3% (5/6) showed evidence of *BAP1* biallelic inactivation. Family history of meningioma was reported in 18.8% (3/16) of patients. Four cases of meningioma were identified during meningioma surveillance imaging, and five cases had recurrences after treatment. Published cases were consistent with the early age of onset, high-grade tumors, and clinical phenotype of tumors.

**Conclusion:**

This study provides additional evidence that high-grade brain and spinal meningiomas are part of the clinical spectrum of *BAP1*-TPDS. Craniospinal imaging surveillance in the *BAP1*-TPDS population should be considered starting around puberty, enabling early detection and management for individuals with *BAP1*-TPDS.

**Supplementary Information:**

The online version contains supplementary material available at 10.1007/s11060-026-05445-2.

## Introduction

*BRCA1*-associated protein-1 (*BAP1*) is a tumor suppressor gene located on chromosome 3p21.1 and serves multiple roles in tumor biology. *BAP1* functions as a deubiquitinating enzyme, playing a crucial role in various cellular processes, including proliferation, DNA repair, differentiation, metabolism, and survival [1]. *BAP1* tumor predisposition syndrome (*BAP1*-TPDS) is an autosomal dominant hereditary cancer syndrome caused by heterozygous germline loss-of-function *BAP1* P/LP variants [2–6]. The syndrome is associated with four main cancers including uveal melanoma, malignant mesothelioma, cutaneous melanoma, and renal cell carcinoma [3, 7, 8]. Affected individuals are also at risk of developing cutaneous *BAP1*-inactivated melanocytic tumors (BIMT), previously described as atypical Spitz tumors. The complete clinical phenotype of *BAP1*-TPDS has not yet been fully characterized and there is ongoing research suggesting the association of several other tumors [4, 6, 8–10]. Proper characterization of the clinical phenotype of the *BAP1*-TPDS is crucial for establishing management guidelines.

In the general adult population, total meningioma incidence is estimated to be approximately 10.82 per 100,000 (0.011%) [11]. Meningioma is the most common primary brain tumor in adults and displays a wide range of clinical and pathological features [12]. Although over 70% of meningiomas are regarded as benign, 5% -20% of patients with meningioma experience recurrences despite receiving maximal standard of care treatments [13, 14]. Higher grade meningiomas (2 and 3) account for 13–16% of meningiomas and are associated with higher rates of recurrence, up to 80% even after complete surgical resection, presenting significant treatment challenges [11, 15]. These tumors frequently lead to more aggressive symptoms and morbidity requiring repeated surgeries and radiotherapy which can substantially impact both quality of life and life expectancy.

Recent studies have identified *BAP1* mutations as a rare but important molecular alteration in meningiomas, potentially associated with poor clinical outcomes includingtumor recurrence, and shortened survival [16]. Though uncommon, loss of BAP1 function —due to either somatic or germline mutations—has been observed in a subset of meningiomas, particularly those with rhabdoid and/or papillary histologic features [16–20]. Rhabdoid and papillary meningiomas are rare subtypes, each accounting for less than 1% of all meningiomas. The diagnosis of these rare subtypes is complicated by their heterogeneity and low incidence, which can lead to variability in histopathologic interpretation and subsequently impact decisions regarding adjuvant therapy [21]. Importantly, preclinical studies suggest that *BAP1*-deficient tumors may be sensitive to targeted therapies, including Enhancer of Zeste Homolog 2 (EZH2) inhibitors and Poly (ADP-ribose) polymerase 1 (PARP1) inhibitors [22, 23] and currently several clinical trials are ongoing [24]. As such, elucidating the genetic drivers—particularly *BAP1* alterations in high-grade meningiomas could meaningfully improve diagnostic precision, prognostic assessment, and treatment strategies for affected patients.

Both The Ohio State University Wexner Medical Center (OSU) and Memorial Sloan Kettering Cancer Center (MSKCC) have specialized BAP1-TPDS clinical programs which incorporate standardized screening protocols for related BAP1 cancers. Currently, the National Comprehensive Cancer Network (NCCN) recommends screening for renal cell carcinoma with MRI abdomen in patients with *BAP1*-TPDS every 2 years beginning at age 30 years [25]. Similarly, our OSU clinic recommends brain and spinal cord craniospinal MRI every 2 years beginning at age 30 years for meningioma surveillance.

The goals of this study were to assess the evidence for association of meningiomas and the clinical phenotype of these tumors in the *BAP1*-TPDS. Our results provide strong evidence that high-grade meningiomas are part of the clinical spectrum of *BAP1*-TPDS.

## Methods

### Retrospective chart review

Our study integrated retrospective chart reviews from two institutional cohorts, OSU and the MSKCC under IRB-approved protocols from October 1st, 2010 to April 21st, 2025, Table 1. At OSU, we included all patients with germline *BAP1* P/LP variants diagnosed, their family members, as well as subjects enrolled in the OSU *BAP1*-TPDS registry, “Frequency and Clinical Phenotype of BAP1 Hereditary Predisposition Syndrome” (NCT04792463 | Registered 3 March 2015 | https://www.clinicaltrials.gov/). Individuals determined to be obligate carriers were also included. Pedigrees were reviewed for reported cases of meningioma, and medical records were obtained to review the pathological diagnoses. For tumors with available archival material (total six tumors, Table 2) the pathology was reviewed by an expert neuropathologist (SSA) to update the tumor grade according to the 2021 CNS WHO guidelines [12]. Also, the expression of BAP1 and proliferative marker Ki67 were assessed. Immunohistochemistry (IHC) was carried out at the OSU Pathology Department, using a prediluted *BAP1* mouse monoclonal antibody (BioSB, clone BSB-109) and Ki-67 antibody. Pretreatment was carried out using Dako Flex High solution for 30 min and detection using the Flex Detection Kit for 20 min using the Omnis autostainer (Agilent, Dako).

**Table 1:** Review of Published Cases of Meningioma in patients with BAP1 Germline Pathogenic Variants

Patient ID	Age^†^	Sex	BAP1 Variant	Meningioma Subtype and Reported WHO Grade	Location	Presentation	Surgery	Recurrence	Alive/Dead	PFS	OS	Personal Cancer History	Family Cancer History^§^
OSU-1	60	F	c.799C>T, p.Gln267*	Mixed, grade N/A	Right parietal brain	N/A	Resection	No	Alive	≥170 mos	≥170 mos	Multiple Basal Cell Carcinomas	Uveal melanoma, benign meningioma, lung adenocarcinoma, mesothelioma (2), cutaneous melanoma, ovarian, kidney, esophageal, colon, stomach, testicular
OSU-2^‡^	24	F	c.1777C>T, p.Gln593*(daughter and mother)	Atypical, Rhabdoid, 2	Extra-axial, prepontine cistern	Symptomatic	Resection and adjuvant radiotherapy for initial tumor, radiation to recurrence.	Yes	Alive	73 mos	137 mos	None	Atypical meningioma, lung, kidney
OSU-3^‡^	58	F		1) Atypical, Rhabdoid/Papillary, 2 2) Meningothelial cyst^┼┼^	1) Left jugular foramen 2) T8/9	1) Symptomatic 2) Asymptomatic, discovered on surveillance scans.	1) Resection and adjuvant radiotherapy. Persistent cystic component being monitored2) Resection	Yes	Alive	34 mos	66 mos	Multiple Basal Cell Carcinomas	
OSU-4	17	M	c.1379C>G, p.Ser460*	1) Rhabdoid, 2 2) Radiographically presumed, grade N/A	1) C6/C7 2) Frontal lobe	1) Symptomatic 2) Asymptomatic, discovered on surveillance scans.	1) Resection 2) Radiotherapy	Yes	Alive	~47 mos	96 mos	None	Skin (unknown type) (2), bladder
OSU-5	27	F	c.877_878del, p.Pro293fs*13	Rhabdoid, 3	Fronto-parietal lobe	Symptomatic	Received adjuvant radiotherapy at time of resection	No	Alive	≥43 mos	≥43 mos	None	Brain, uveal melanoma, squamous cell carcinoma, uveal melanoma, breast
OSU-6	59	F	c.660-2A>G	Atypical, subtype N/A, 3	N/A	N/A	N/A	Unknown	LTF	N/A	≥58 mos	None	Cutaneous melanoma, mesothelioma, breast, colon, uveal melanoma, multiple myeloma
OSU-7	36	F	c.1717delC (p.Leu573pfs*3)	Rhabdoid, 3	Right occipital lobe	Symptomatic	Initial treatment with radiotherapy, recurrence treated with surgical excision and adjuvant radiation	Yes	LTF	132 mos	136 mos	None	Cholangiocarcinoma (2), multiple basal cell carcinomas, vulvar, cutaneous melanoma, breast, mesothelioma (3), colon (2)
OSU-8	46	M	c.37+1G>T	Papillary, 3	Anterior cranial fossa	Symptomatic	Resection	No	Alive	44 mos	44 mos	Basal Cell Carcinoma	Cervical, skin (unknown type), breast
OSU-9	62	M	EX12_3'UTRdel	N/A, 2	Anterior clinoid and left cerebellar hemisphere	Asymptomatic, discovered on surveillance scans	Resection and radiotherapy to residual disease	No	Alive	19 mos	19 mos	Renal Cell Carcinoma	Cutaneous melanoma, prostate, breast, pancreatic (3), throat, renal cell carcinoma (2)
OSU-10	60	F	c.1891-1G>A	Radiographically presumed, grade N/A	Brain	Asymptomatic, discovered on surveillance scans	No intervention	No	Alive	≥43 mos	≥43 mos	Renal Cell Carcinoma, pancreatic neuroendocrine cancer, basal cell carcinoma	Cutaneous melanoma, multiple basal cell carcinomas, breast (2), uterine
OSU-11	40	F	c.37+1G>T	1) Atypical, 1 2) Radiographically presumed, grade N/A	1) Left frontal 2) Right parietal	1) Symptomatic 2) Asymptomatic, discovered on surveillance scans	1) Resection 2) No intervention, monitored bi-annually	Yes	Alive	12 mos	40 mos	Renal Cell Carcinoma	Breast (2), cutaneous melanoma, thyroid
OSU-12	N/A	F	EX10_11del	Radiographically Presumed, grade N/A	Brain	Asymptomatic, discovered on surveillance scans	N/A	No	Alive	≥72 mos	≥72 mos	Breast, Renal Cell Carcinoma, Cholangio- carcinoma	Uveal melanoma, breast, mesothelioma, cutaneous melanoma, renal cell carcinoma
OSU-13	17	F	c.1153C>T, p.Arg385*	Radiographically Presumed, grade N/A	Parieto-occipital	N/A	Interval resolution without direct intervention	No	Alive	≥106 mos	≥106 mos	Uveal melanoma	Lung (2), bladder, esophageal
MSKCC-14	32	M	c.1675_1684delACAGGCCTGC	N/A	N/A	N/A	Resection	Unknown	N/A	N/A	N/A	Non-small cell lung, mesothelioma	Pancreatic, cutaneous melanoma
MSKCC-15	59	F	c.1254T>A, p.Tyr418*	Atypical, grade N/A	Left frontoparietal region	Symptomatic	Resection	Unknown	N/A	N/A	N/A	Mesothelioma, kidney, breast	Bladder, peritoneal mesothelioma vs. ovarian carcinoma
MSKCC-16	71	F	c.1203T>G,p.Tyr401*	Rhabdoid, 1	Left orbital roof/floor of anterior cranial fossa	Asymptomatic, discovered on surveillance scans	Resection	Unknown	N/A	N/A	N/A	Basal cell carcinoma	Pancreatic, squamous cell carcinoma, basal cell carcinoma, uveal melanoma, brain, liver

†Age in years at diagnosis. ‡Patient 2 is the daughter of Patient OSU-3, they are included in Hu et al, 2022 [27] and Prasad et al, 2021 [28]. §1^st^ and 2^nd^ degree relatives were counted.^┼┼^ Meningothelial cyst was not included in the total meningioma numbers. Abbreviations: WHO, World Health Organization; OSU- Ohio State University; MSKCC, Memorial Sloan Kettering Cancer Center; N/A, not available, LTF, lost to follow-up

**Table 2:** Pathology characteristics of meningiomas with available tissue for reassessment

Patient ID	Morphology Rhabdoid and Papillary	Brain Invasion	Ki67	Mitotic Count (per 10 HPF)	BAP1 IHC/Genotyping	Reported Grade	WHO Grade 2021
OSU-1	Both	Negative	N/A	3	Loss of nuclear expression/LOH	N/A	1
OSU-2	Rhabdoid	Negative	15%	3	Loss of nuclear and cytoplasmic expression	2	2
OSU-3	Both	Negative	10%	1	Loss of nuclear and cytoplasmic expression	2	1
OSU-4	Rhabdoid	Negative	8%	8	Loss of nuclear and cytoplasmic expression	2	2
OSU-5	Rhabdoid	Negative	8%	7	Loss of nuclear and cytoplasmic expression	3	2
OSU-9	Neither	Positive	2%	1	Positive nuclear expression	2	2

Abbreviations: OSU, Ohio State University; IHC, immunohistochemistry; WHO, World Health Organization; LOH, loss of heterozygosity; N/A, Not available

At MSKCC, a retrospective chart review was conducted with all patients with germline BAP1 P/LP variants who had genetic consultation at the institution’s Clinical Genetics Service or underwent genetic testing through MSK-IMPACT. All patients were assigned a study ID code and clinical data regarding the patient’s age, sex, clinical presentation, and meningioma characteristics including pathology were obtained from the records.

### Literature review

A review of published literature was conducted, focusing on identifying documented cases of germline *BAP1* P/LP variants in meningiomas (Supplementary Fig. 1). Published articles in PubMed and Scopus were reviewed for cases of patients with meningiomas and germline BAP1 P/LP variants to establish an association between BAP1-TPDS and meningiomas. Meningioma morphology, WHO grade, BAP1 P/LP variants, and clinical characteristics were recorded. It is important to note that two of the patients discussed in the literature review also belong to the OSU institutional cohort.

## Results

### Demographic characteristics

The OSU cohort had 13 cases of meningioma in a cohort of 195 patients with BAP1-TPDS (13/195), while the MSKCC had 3 patients diagnosed with meningioma in a cohort of 42 with Bap1-TPDS (3/42), for a total of 16/237 cases of meningioma in patients with germline *BAP1* P/LP variants (Table 1). These cases included 13 with confirmed pathological diagnosis and 3 cases with radiologically presumed. One patient had a meningothelial cyst found along the spinal meninges a few years following the diagnosis and resection of her initial cranial meningioma. This patient and her daughter have been described previously [26, 27]. The mean age at presentation in this cohort is 44.5 years old (median = 46). Notably, the mean age at presentation for meningioma in the general population is 66 years old [11]. 75% of patients were females while only 25% patients were males. While most tumors were identified in symptomatic patients, four asymptomatic patients had tumors found during surveillance imaging for their known BAP1 P/LP variant status. During pedigree review, we identified an obligate carrier with a reported unspecified brain tumor; medical chart history for this deceased individual could not be collected to confirm diagnosis, therefore this patient was not included in the meningioma totals.

Two patients with meningioma were related (mother and daughter), however, having a family member with meningioma was uncommon. Only one other patient, besides the mother and daughter (3/16), reported having a 1st or 2nd degree relative with meningioma. However, family history of other *BAP1* related cancers was typical with 81.3% (13/16) patients reported having one or more 1st or 2nd degree relatives with cancers associated with BAP1-TPDS, including cutaneous melanoma (7/16), uveal melanoma (5/16), mesothelioma (5/16), kidney cancer (4/16), basal cell carcinoma (3/16), and other cancers (Table 1). Among the patients with meningioma, 62.5% (10/16) had a separate primary tumor including kidney cancer (5/16), basal cell carcinomas (3/16), mesothelioma (2/16), breast cancer (1/16), cholangiocarcinoma (1/16), non-small cell lung cancer (1/16), uveal melanoma (1/16), and neuroendocrine tumor (1/16).

### Tumor characteristics, histopathology and clinical outcome

**Fig. 1:**
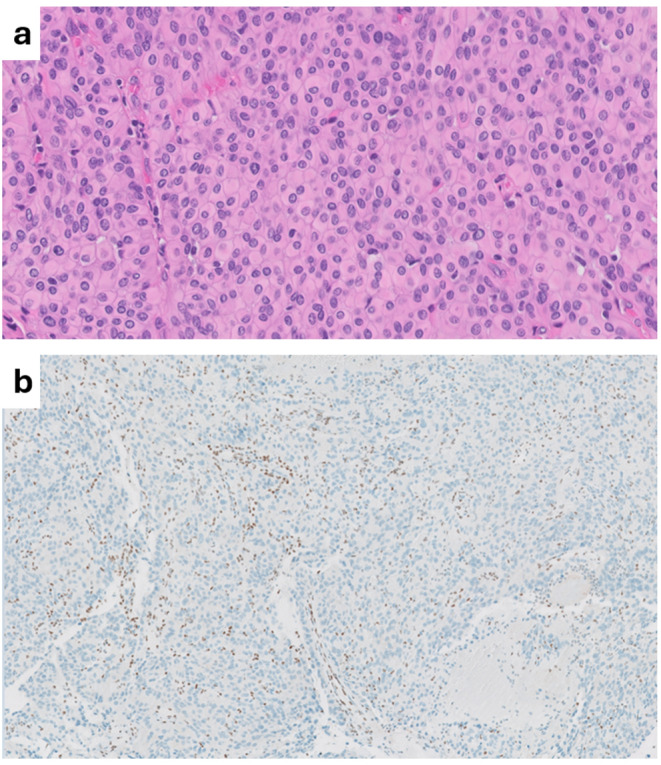
**a**) A case with rhabdoid morphology, (OSU-2), 40x magnification, **b**) BAP1 loss in a case with WHO grade I rhabdoid/papillary, (OSU-3) with BAP1 loss in tumor cells. Note positive staining in inflammatory cells and non-tumor blood vessels, 10x magnification

A majority of meningiomas, 81.3%, were found in the cranium (13/16); 1 was found along the spinal meninges, and 2 were of unknown primary sites (Table 1). 23% (3/16) of meningiomas were radiographically presumed or otherwise did not have available pathology. For the pathology confirmed meningiomas, 38.5% (5/13) had rhabdoid morphology, 30.8% (4/13) had atypical pathology with unspecified histologic subtype, 7.7% (1/13) had papillary, 7.7% (1/13) had both papillary and rhabdoid, and 7.7% (1/13) had transitional/mixed morphology (Fig. 1). The morphology was not available for one patient (Table 1). Furthermore, we identified cystic lesions of the meninges in one patient, OSU-3, who was diagnosed with a brain meningioma and later had a cystic recurrence of her meningioma after surgical resection and adjuvant radiotherapy. During routine screening of her brain and spine, she was later found to have a meningothelial cyst at C7-C8 separate from the initial brain meningioma.

WHO grading was available in 10 tumors, 20% (2/10) were WHO grade 1, 40% (4/10) were grade 2, 40% (4/10) were grade 3. A total of 80% (8/10) of the tumors were considered high-grade. We were unable to confirm WHO grade for 6 of the tumors due to lack of tissue or inability to obtain tissue or pathology reports from outside institutions. Given the changes in WHO grading of meningioma in 2021, we carried out reassessment of six tumors with available archival material (OSU-1, OSU-2, OSU-2, OSU-4, OSU-5, and OSU-9).

In the OSU cohort, the median follow up duration was 66 months (range 19–170 months). local recurrence of previously treated tumors was reported in five (41,7%), three of which had rhabdoid pathology. The fourth recurrence was noted to be a cystic recurrence, and the fifth did not have available pathology. The median duration for progression free survival was 47 months (range 12–170 months). For the OSU cohort at the time of last follow up 11 patients were alive and two were lost for follow up. 

For the six reanalyzed tumors our assessment down-graded two tumors, Table 2. The proliferative index for these patients ranged from 2% to 15% (Table 2). For four patients, the mitotic count was less than 4 mitoses per ten high power fields. Prior studies have reported that the absence of BAP1 protein expression by immunohistochemistry correlates over 88% with true molecular BAP1 loss and/or mutations in tumor tissue [28, 29]. Somatic BAP1 loss was confirmed via immunohistochemistry in 5 patients from the OSU cohort; BAP1 expression was preserved in the tumor tissue of one patient (OSU-9).

Clinical molecular analysis was available on one tumor (OSU-9), while in two tumors research exomes were reported in a previous study (OSU-2 and OSU-3) [26]. In all three tumors no mutation was reported in CDKN2A/B, TERT, TRAF7, and NF2.

### Literature review

The comprehensive literature review aimed to ascertain the prevalence and characteristics of meningiomas lacking *BAP1* expression in published case reports. We identified a total of 13 patients with BAP1 P/LP variants and pathology confirmed meningioma (Table 3, Supplementary Fig. 1). Consistent with our cohort, the mean age at diagnosis was 46.7 years old (median = 56). 84.6% (11/13) of patients were females, 7.8% (1/13) of patients were male, and the gender of 7.8% (1/13) patients was not reported. Six patients were noted to have clinical symptoms that led to the discovery of their lesions, whereas the others were not specified.

**Table 3:** Literature Review of Published Cases of Meningioma in patients with BAP1 Germline Pathogenic Variants

Reference	Age	Sex	BAP1 Variant(s)	Meningioma Subtype and WHO Grade	Location	Presentation	Evidence of biallelic inactivation	Intervention and outcome	Personal cancer history	Family cancer history
[18]	59	F	p.G220_ splice (exact mutation N/A)	Rhabdoid, III	Left frontal	N/A	Loss of nuclear BAP1 in tumor	Unknown treatment, lost to follow-up	Unknown	Mesothelioma
	53	M	c.519T>G (p.Tyr173*)	Rhabdoid, III	Left parietal	N/A	Loss of nuclear BAP1 in tumor	Resection and adjuvant radiation, three recurrences	Unknown	Unknown
[19]	12	F	c.1174C>T, p.Gln392*	Rhabdoid, III	Right tentorium	Symptomatic	Loss of nuclear BAP1 in tumor	Resection and adjuvant radiation, multiple recurrences	None	Leukemia
[30]	NA	F	c.1938T>A, p.Tyr646* (sisters)	N/A	N/A	N/A	N/A	N/A	Mesothelioma (pleural), basal cell carcinoma	Mesothelioma (n=2), uveal melanoma (n=1), breast (n=1), cutaneous melanoma (n=2), basal cell carcinoma (n=1)
	NA	F		N/A	N/A	N/A	N/A	N/A	Mesothelioma (peritoneal)	
[27]	64	F	c.778C>T (mother and daughter)	Radiographically presumed	Left frontoparietal convexity	Symptomatic	Loss of nuclear BAP1 in mesothelioma tumor	Deceased prior to pathologic confirmation	Multiple basal cell carcinomas, pleural mesothelioma	Mesothelioma (n=3), renal cell carcinoma (n=2), bladder (n=1), melanoma (n=1)
	42	F		Papillary, III	Right sphenoid sinus.	Symptomatic	N/A	Resection, adjuvant radiation, deceased ~ 48 months after diagnosis	Pleural mesothelioma	
	70	F	c.1717delC	Rhabdoid, III	Adjacent to right sylvian fissure	Symptomatic	Loss of nuclear BAP1 in tumor	Resection, deceased < 15 months after resection	Pleural mesothelioma, multiple basal cell carcinomas, breast cancer, colon cancer, lung cancer	N/A
	24	F	c.1777C>T, p.Gln593(daughter and mother)^†^	Rhabdoid, II	Extra-axial, prepontine cistern	Symptomatic	Loss of nuclear BAP1 (Table 2)	Resection and adjuvant radiotherapy,stable disease	None	Mesothelioma (n=1), renal cell carcinoma (n=1)
	58	F		N/A, Grade II^‡^	Not specified (see Table 1)	Symptomatic	Loss of nuclear BAP1 (Table 2)	Not specified (see Table 1)	Not listed (see Table 1)	
[31]	19	F	c.1478_1479delCA (p.Thr493Argfs*5)	Rhabdoid, II	Brain, unspecified	Incidental (MRI for post-concussive headache)	Copy number loss affecting entire chromosome 3	N/A	N/A	N/A
[33]	57	M	c.118C>T (p.Gln40*)	Papillary, III	Intraventricular	N/A	Focal copy number loss	Resection, second tumor appeared after 12 months, intradural adjacent to L5	Uveal melanoma, basal cell carcinoma	N/A
[32]	56	F	Whole gene deletion	Rhabdoid, III	Sphenoid wing and temporalconvexity (separate tumors)	N/A	Loss of nuclear BAP1 in sister’s hepatoid tumor	Two separate recurrent tumors with multiple excisions	Uveal melanoma, basal cell carcinoma, mesothelioma	Basal cell carcinoma (n=5), hepatoid carcinoma of the pancreas (n=1), stomach (n=1), cutaneous melanoma (n=1)

†Patient 4 and 5 from Hu et al are also included in our institutional cohort as OSU-2 and -3, and are described by Prasad et al, 2021 [26]. ‡At OSU, this tumor was classified as a WHO Grade I meningioma with papillary and rhabdoid features. Abbreviations: WHO, World Health Organization; N/A, not available

76.9% (10/13) patients had intracranial meningiomas. The location of the meningioma was not specified in 23.1% (3/13) patients. Of the 10 patients with reported histopathology, 60% (6/10) were found to have meningiomas with predominantly rhabdoid morphology, 20% (2/10) patients were found to have a meningioma with papillary morphology, and 20% (2/10) patients were found to have both rhabdoid and papillary morphology [16–32]. In publications where mitotic activity was measured in tumor sections, the mitotic range varied from very low overall (1 per 10 high-power fields [HPFs]) to very high (15 per 10 HPFs) [16, 19, 33]. Similarly, in publications where Ki67 antibody immunostaining was applied to tumor sections, the proliferative index ranged from 3% to 15% [19, 33].

## Discussion

Recognizing the need for meningioma screening in individuals with germline *BAP1* P/LP mutations is an emerging priority in the management of *BAP1*-TPDS. Most meningiomas are slow-growing and benign [34]. However, recent case studies have reported that meningiomas with somatic and germline *BAP1* loss are particularly aggressive, and are associated with significant morbidity including seizure, focal neurologic deficits, altered mental status, weakness, and hydrocephalus [15–20]. Our study highlights the high prevalence of higher grade recurring meningiomas in a subset of patients with germline *BAP1* P/LP variants. This observation prompts a re-evaluation of the tumor spectrum associated with *BAP1*-TPDS and underscores the potential importance of including meningiomas within the surveillance spectrum for these individuals.

### Frequency of meningiomas in BAP1-TPDS

Based on early data, our group’s prior estimated frequency of meningioma in *BAP1*-TPDS patients was 1.7% [35]. In the current study, evaluating both the OSU and MSKCC cohorts, the frequency of meningioma in BAP1-TPDS was 6.7% (13/195) and 7.1% (3/42), respectively, with a combined frequency of 6.8% (16/237) supporting the high frequency in our previous study [8]. Collectively, these data represent a much higher prevalence among BAP1-TPDS patients than previously thought and further supports the published literature of increased incidence rates.

### Unique high grade histopathology subtypes of BAP1-TPDS associated meningiomas

Meningiomas associated with *BAP1*-TPDS have most commonly been found to have rhabdoid morphology. Interestingly, in our cohort, there were patients found to have papillary, rhabdoid, or a combination of both rhabdoid and papillary morphologies. These data are further supported by studies by Shankar et al., in which 2 cases of germline BAP1-mutated meningiomas were found to have both papillary and rhabdoid morphology [16]. A recent report by Sievers et al. found that 81% of somatic BAP1-altered meningiomas in their cohort had predominantly rhabdoid morphology but also reported papillary components, less commonly meningothelial and other mixed morphologies [36]. Rhabdoid and papillary morphology are both unique subtypes which used to be classified as higher grade features [16]. However, the 2021 WHO Classification of CNS Tumors no longer classifies meningiomas with rhabdoid and papillary subtype as high-grade tumors solely based on architecture and requires additional high-grade features for the designation of WHO grade 3 [12]. Even in the absence of these higher-grade features, BAP1 loss in these tumors is associated with more aggressive behavior including reduced time to recurrence, warranting close follow-up [37]. This suggests that mutations in BAP1 may not be limited to just rhabdoid morphology, and therefore the phenotype of meningioma in *BAP1*-TPDS should be expanded to include other subtypes including papillary, transitional and meningothelial [36]. Additional follow-up and studies of progression free and overall survival would be helpful to evaluate the clinical course and aggressiveness of meningioma in BAP1-TPDS. The incorporation of BAP1 in meningioma molecular testing is crucial for not only diagnosis and grading, but as predictor of high grade and possibly more aggressive tumors [36]. Given the rarity of BAP1-mutated meningiomas and the relative high frequency of germline alterations in these cases [16], reflex germline testing is recommended.

### Meningioma treatment and recurrence

In cases where the tumor is accessible, gross total resection followed by radiotherapy is the preferred primary treatment course for grade 2 or 3 meningiomas [38]. In the current study, all patients with available surgical and pathological data had undergone primary surgical resection and some received adjuvant radiation. For the patients with available follow up data, five patients had a local recurrence 41.7% (5/12). One of our patients who was diagnosed with an intracranial meningioma was found to have a cystic recurrence of meningioma after surgical resection and adjuvant radiotherapy (patient OSU-3). She later was found to have a meningothelial cyst at C7-C8 during routine screening, which had not been described in earlier case reports of her and her daughter [26, 27]. In patient OSU-7, the initial tumor grade is unknown, however she underwent resection of her local recurrence, and the final WHO grade (prior to 2021) of this tumor was Grade 3 with more than 10 mitoses per 10 high power field and a 50% Ki67 labeling index. At least two patients presented with multiple tumors in different anatomical locations.

In the literature review, patients with higher grade meningiomas had multiple recurrences [16, 19, 32, 33]. One patient was reported as having a metastasis which was distant from the initial meningioma [33]. The spectrum of diseases associated with BAP1-TPDS may include risk of recurrence or of cystic lesions of the meninges. Further work is needed to study recurrence rates and best treatment options for BAP1-inactivated meningiomas.

### Screening recommendations for BAP1-TPDS patients

Standardizing screening for the *BAP1*-TPDS patient population is important; absence of formalized screening guidelines could lead to higher risk of delayed diagnosis and associated morbidity in patients with *BAP1*-TPDS. Our study showed that *BAP1*-TPDS patients are at a higher risk of developing meningioma which are diagnosed at an earlier age than the general population, emphasizing a need for standardized meningioma screening. Only 23.1% (*n* = 3/13) patients from the OSU cohort reported having a 1st or 2nd degree relative with meningioma, indicating that their meningioma did not involve familial clustering. Many patients did have personal cancer history, and most also had a family history of cancers associated with *BAP1*-TPDS. Our results support regular screening for meningioma in *BAP1*-TPDS patients irrespective of the family history of meningiomas.

Other cancers associated with *BAP1*-TPDS have screening guidelines: for example, to detect uveal melanoma, patients are recommended to have a yearly dilated eye exam beginning at age 11, which is 5 years earlier than the youngest patient diagnosed with uveal melanoma [6]. Remarkably, 25% of the cases in our cohort (4/16) and in the literature review (3/12) were diagnosed with meningioma prior to age 30 and as young as age 17. These findings suggest that biennial craniospinal imaging should begin prior to age 30, perhaps around puberty. If suspicious areas are noted on MRI, GA-68 DOTATATE PET scans are an option to help evaluate areas concerning for meningioma, as this technology can find small and multifocal meningiomas [39]. Clinicians should also take into consideration the psychological impact of screening, healthcare costs, and potential implications of detecting incidental findings.

### Study limitations

The OSU cohort is enriched in patients with uveal melanoma and patients with personal/family cancer histories consistent with *BAP1*-TPDS, whereas MSKCC accrues patients with diverse cancers. Further research may allow for more robust data sets and more diverse manifestations of *BAP1*-TPDS to be detected and further refine our understanding of disease prevalence. Due to the rarity of BAP1-TPDS, the cohort is relatively small, limiting the strength of the data. As more patients are diagnosed, their data will be added to our study for future publications. In addition, there was a lack of clinical follow-up information on some patients; and only part of the cohort was screened for meningioma with standardized craniospinal imaging protocols, as many patients came from outside institutions. Longer term follow up will be captured in a future publication. Some of the lesions in our participants are radiographically presumed or they did not have available tissue or pathology reports as they obtained their care at outside institutions more than 10 years ago. Without tissue diagnosis, we could not confirm the etiology of the lesions with complete certainty. This also prevents us from ascertaining details regarding histopathologic cell type and BAP1 loss in the lesions. Moreover, molecular testing was only performed in a small percentage of the cohort. The presence of *CDKN2A/B*, TERT, NF2, and TRAF7 therefore, was not available for a majority of the cohort. These mutations may have impact on grading of the tumors and are important for prognosis and outcomes. Testing of future patients in our cohort may allow for better understanding of the role of *CDKN2A/B* and TERT in meningiomas in this population.

## Conclusion

In conclusion, our findings underscore the value of considering meningioma screening in individuals with germline *BAP1* mutations. We observed a 6.8% prevalence of meningioma in two large cohorts of *BAP1*-TPDS patients. By utilizing a proactive approach to surveillance imaging, we allow for the potential for early detection, timely intervention, and personalized treatment strategies with the chance to improve outcomes for these individuals with *BAP1*-TPDS who have predisposition to the development of meningioma. Continued research into the clinical and molecular characteristics of meningiomas in the context of *BAP1*-TPDS will further guide the development of effective surveillance strategies and personalized therapeutic interventions for these individuals.

## Supplementary Information

Below is the link to the electronic supplementary material.

Supplementary Material 1

## Data Availability

All original data generated in support of this research will be made available at reasonable request to the corresponding author.
