# Supplementary figures and images for: High frequency and unique subtypes of meningioma in patients with BAP1 tumor predisposition syndrome
Source: J Neurooncol. 2026 Feb 11;176(3):207. doi: 10.1007/s11060-026-05445-2 (PMC12894157; doi:10.1007/s11060-026-05445-2)

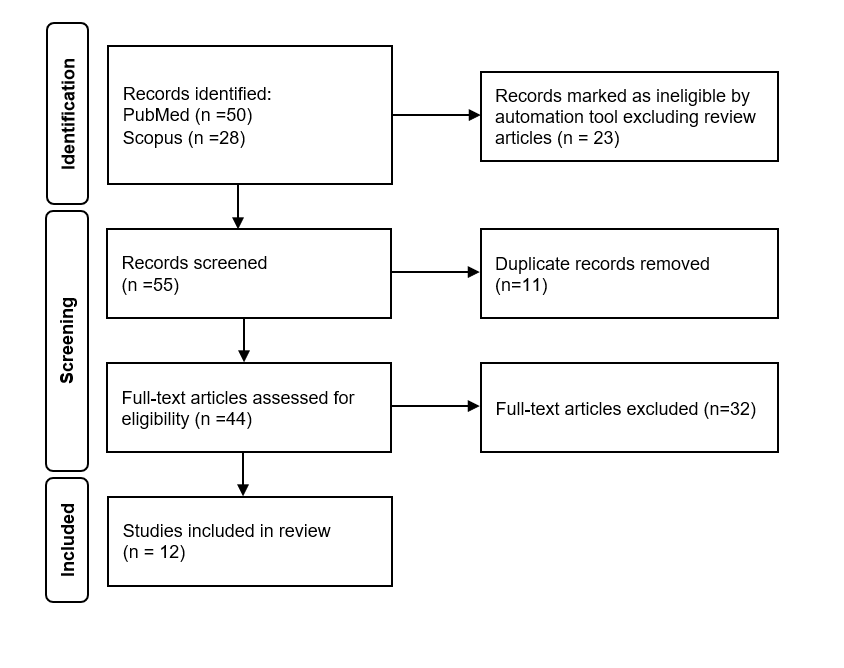

Supplement: Supplementary file 1 — Supplementary Material 1 [file 11060_2026_5445_MOESM1_ESM.png]
